# IGF-1 mediated Neurogenesis Involves a Novel *RIT1*/Akt/Sox2 Cascade

**DOI:** 10.1038/s41598-017-03641-9

**Published:** 2017-06-12

**Authors:** Sajad Mir, Weikang Cai, Shaun W. Carlson, Kathryn E. Saatman, Douglas A. Andres

**Affiliations:** 1 Department of Molecular and Cellular Biochemistry, University of Kentucky College of Medicine, Lexington, Kentucky 40536-0509 USA; 2 Joslin Diabetes Center, Harvard Medical School, Boston, Massachusetts 02215 USA; 3 Spinal Cord and Brain Injury Research Center and Department of Physiology, University of Kentucky College of Medicine, Lexington, Kentucky 40536-0509 USA; 4 Department of Neurosurgery, Safar Center for Resuscitation Research, University of Pittsburgh, Pittsburgh, Pennsylvania 15260 USA

## Abstract

Insulin-like growth factor 1 (IGF-1) is known to have diverse effects on brain structure and function, including the promotion of stem cell proliferation and neurogenesis in the adult dentate gyrus. However, the intracellular pathways downstream of the IGF-1 receptor that contribute to these diverse physiological actions remain relatively uncharacterized. Here, we demonstrate that the Ras-related GTPase, *RIT1*, plays a critical role in IGF-1-dependent neurogenesis. Studies in hippocampal neuronal precursor cells (HNPCs) demonstrate that IGF-1 stimulates a RIT1-dependent increase in Sox2 levels, resulting in pro-neural gene expression and increased cellular proliferation. In this novel cascade, RIT1 stimulates Akt-dependent phosphorylation of Sox2 at T118, leading to its stabilization and transcriptional activation. When compared to wild-type HNPCs, *RIT1* ^*−*/*−*^ HNPCs show deficient IGF-1-dependent Akt signaling and neuronal differentiation, and accordingly, Sox2-dependent hippocampal neurogenesis is significantly blunted following IGF-1 infusion in knockout (*RIT1* ^*−*/*−*^) mice. Consistent with a role for RIT1 function in the modulation of activity-dependent plasticity, exercise-mediated potentiation of hippocampal neurogenesis is also diminished in *RIT1* ^*−*/*−*^ mice. Taken together, these data identify the previously uncharacterized IGF1-*RIT1*-Akt-Sox2 signaling pathway as a key component of neurogenic niche sensing, contributing to the regulation of neural stem cell homeostasis.

## Introduction

Adult neurogenesis is a dynamic process in which new neurons are generated from neural stem/progenitor cells (NPCs) through the carefully orchestrated regulation of proliferation and neuronal fate determination. Adult born neurons have the ability to functionally integrate into the hippocampal circuitry and have been found to contribute to select types of hippocampal-dependent learning and memory tasks^1^. A critical feature of adult neurogenesis is its regulation by diverse physiological, pathological, and pharmacological stimuli, including exercise, aging, traumatic brain injury, epilepsy, and antidepressants^2^. NPCs are normally quiescent, but once activated are capable for self-renewal to maintain the neural stem cell population and to produce dividing progenitors capable of differentiating into neurons^3–5^. Significantly, the maintenance and proliferation of adult NPCs, and the differentiation, migration, and maturation of adult born neurons, are known to be regulated by extracellular growth factor signaling pathways^6, 7^. Growth factor signaling is known to control a variety of intracellular regulatory mechanisms within NPCs, including transcription factors and epigenetic regulators that serve to finely coordinate gene expression during neurogenesis^2^. For example, the transcription factor Sex-determining region Y related HMG box 2 (Sox2) has well-established roles in maintaining stem cell/progenitor cell properties in diverse cellular populations^8, 9^, including neuronal stem cell self-renewal and neurogenesis^10^.

Insulin-like growth factor-1 (IGF-1) signaling has been implicated in the regulation of adult neurogenesis^11–13^. For example, conditional deletion of the IGF-1 receptor gene (*Igf1r*) results in an almost complete loss of the dentate gyrus (DG) in mice^14^ while exogenous IGF-1 promotes enhanced HNPC proliferation^15, 16^, and directs the generation of mature granule cells^15–18^. These effects rely on the ability of activated IGF-1R to activate diverse intracellular regulatory cascades, including the ERK MAP kinase and PI3-kinase/Akt pathways^19, 20^. In particular, Akt signaling has been reported to play a central role in IGF-1-mediated HNPC proliferation and neuronal differentiation^21–24^. IGF-1/Akt signaling is also known to control neuronal transcription factor activity^22, 25^, however the nature of the specific transcriptional pathway(s) involved in IGF-1 dependent neural stem cell activation remain incompletely characterized.

Running is a particularly potent instigator of hippocampal neurogenesis with evidence from human and animal research indicating that exercise improves hippocampal function, synaptic plasticity, learning, and modulates depression^26^. Lineage tracing studies^27^ have demonstrated that voluntary exercise increases the number of Sox2^+^ neural stem cells in the DG, and importantly, pharmacological inhibition of Akt has been found to inhibit exercise-mediated enhancement of adult hippocampal neurogenesis^28, 29^. Since, exercise increases the availability of several classes of growth factors, including IGF-1^30^, this work suggests a link between physiological/growth factor cues, Akt signaling, and the regulation of neurogenesis. The discovery of signaling pathways that enhance adult neurogenesis may lead to therapeutic strategies for improved memory function due to aging or following injury. Therefore, it is important to develop a detailed molecular understanding of these cascades.

The Ras-related GTPase, *RIT1*, is expressed throughout the central nervous system and within the developing brain^31–33^. Despite its widespread expression, *RIT1* function remains incompletely characterized^34^. We have previously demonstrated that RIT1 serves as a central regulator of stress-activated MAPK activity and pro-survival signaling^35^. In keeping with a role in directing survival signaling, adult born immature neurons lacking RIT1 display significantly increased rates of trauma-induced loss following contusive brain injury^36^. Surprisingly, *RIT1* knockout (*RIT1* ^*−*/*−*^) mice also exhibit a significant delay in the innate restoration of neurogenesis following brain injury^36^, suggesting that RIT1 controlled signaling contributes to the period of enhanced hippocampal neurogenesis observed following brain insult^37^. More recently we identified somatic *RIT1* mutations in a subset of human lung adenocarcinomas, and found that ectopic expression of mutated RIT1 in cell culture activates Akt signaling and promotes cellular transformation^38^. Importantly, expression of oncogenic RIT1 also stimulates the Akt-dependent proliferation of HNPCs^39^. Here, we demonstrate the physiological importance of *RIT1* function by establishing a role for RIT1 in both IGF-1 dependent control, and exercise-enhanced stimulation, of hippocampal neurogenesis. Using *in vivo* and *in vitro* genetic strategies to manipulate RIT1 function, we find that IGF-1 stimulates a RIT1-Akt-Sox2 signal transduction cascade in HNPCs that leads to increased neurogenesis. In this pathway, RIT1-Akt activity results in phosphorylation of Sox2 at T118, to increase Sox2 transcriptional activity, enabling IGF-1 directed activation of the neurogenic program.

## Results

### Loss of RIT1 alters exercise-induced neurogenesis

**Figure 1:**
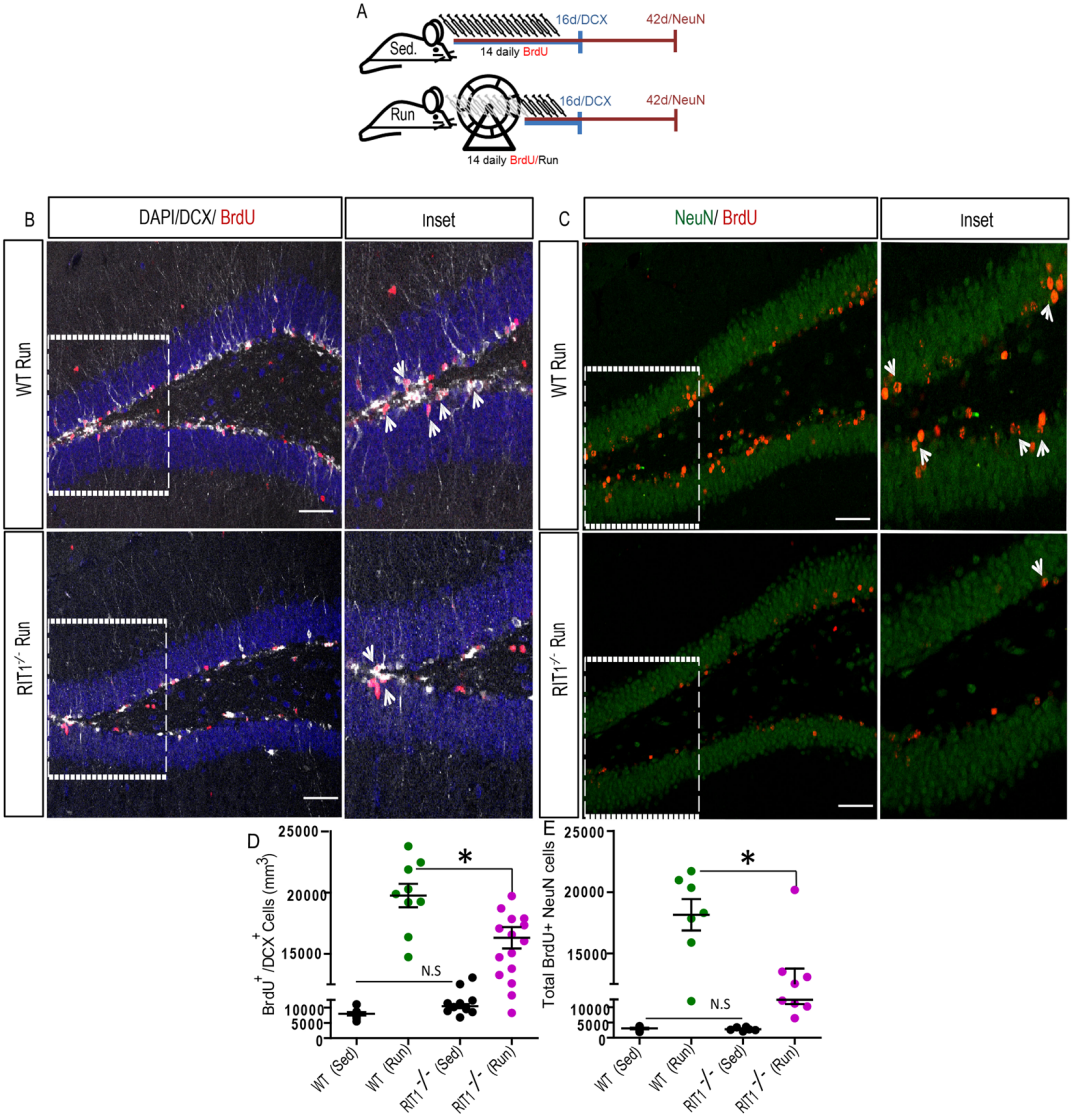
Adult neurogenesis in *RIT1* ^*−*/*−*^ and WT littermates housed under running conditions. (**A**) Schematic of experimental design (see methods for details). WT and *RIT1* ^*−*/*−*^ mice were injected daily with BrdU (50 mg/kg, i.p) for 14 days while housed in either sedentary or running cages and either analyzed for newborn neuroblasts (DCX^+^/BrdU^+^) (day 16) or the generation of mature neurons (NeuN^+^/BrdU^+^) (day 42). Representative coronal hippocampal sections from the dentate gyrus of (**B**) 16d mice immunostained for DCX (*white*, pseudocolor), BrdU (*red*, white *arrowheads*) and DAPI, or (**C**) 42d mice co-immunostained with NeuN (green) and BrdU (*red*; white *arrowheads*). The inserts are at higher magnification. The arrow heads indicate either newborn neuroblasts (**B**) or mature neurons (**C**). Scale bar, 20 μm. (**D**) Quantitative analysis of newborn immature hippocampal neurons (BrdU^+^/DCX^+^) following 16 days of running or sedentary housing (*p < 0.01, two-way ANOVA). (**E**) Quantitative analysis of BrdU^+^ mature neurons (NeuN^+^) at day 42 (*p < 0.01, two-way ANOVA). The results are presented as mean ± SEM (WT, n = 17; *RIT1* ^*−*/*−*^, n = 23).

We have shown that *RIT1* deficiency alters hippocampal neurogenesis following traumatic brain injury by delaying the post-concussive recovery of immature neurons without affecting basal rates of neurogenesis^36^. However, whether RIT1 contributes to the regulation of adult neurogenesis more broadly in response to physiological stimuli is unknown. Importantly, RIT1 is known to be expressed in HNPCs, supporting a potential role for RIT1 in the regulation of neural progenitor function^39^. To assess the role of RIT1 in voluntary exercise-enhanced hippocampal neurogenesis^40^, RIT1 knockout (*RIT1* ^*−*/*−*^) (n = 17) and wild-type (n = 23) mice were randomly assigned to either sedentary or running groups (running wheels were preinstalled in the housing cages permitting voluntary exercise), and the impact of RIT1 loss on exercise-enhanced neurogenesis assessed at either day 16 or 42 (Fig. 1A). Both wild-type and *RIT1* ^*−*/*−*^ mice in the runner groups ran an average distance of ~10 kilometers a day with no inherent difference arising from *RIT1* deficiency (wild-type: 10.25 ± 1.14 km/d; *RIT1* ^*−*/*−*^: 10.10 ± 0.59 km/d, p = 0.86, n = 3). During the trial, mice received one daily intraperitoneal BrdU injection (50 mg/kg) for the first 2 weeks, to label proliferating neuroblasts (BrdU^+^/DCX^+^ cells) (analysis day 16) (Fig. 1B) and maturing neurons (analysis day 42; 1 month post-BrdU chase) (BrdU^+^/NeuN^+^) (Fig. 1C). While lineage tracing detected approximately equivalent numbers of BrdU labeled proliferating neuroblasts (p > 0.05) and mature neurons (p > 0.05) in sedentary housed mice (p > 0.05), *RIT1* ^*−*/*−*^ mice displayed a significantly lower density of proliferating neuroblasts (*p < 0.01), and neurons that matured from these neuroblasts following running exercise than wild-type controls (*p = 0.01) (Fig. 1D,E). These data suggest that RIT1 signaling contributes to the proliferation and neuronal differentiation following voluntary exercise.

### RIT1 contributes to IGF-1 dependent neurogenesis

**Figure 2:**
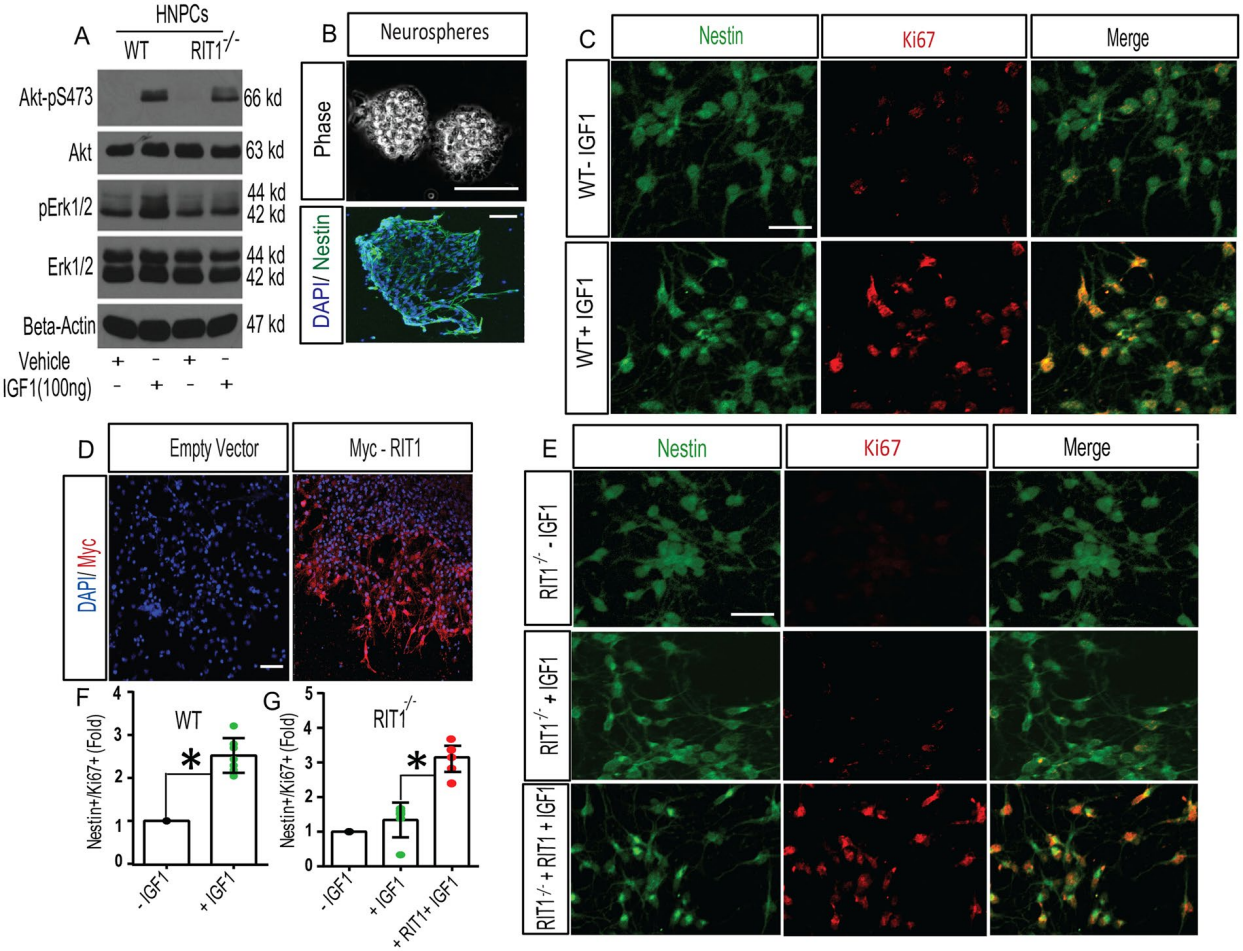
*RIT1* loss disrupts IGF-1-mediated *in vitro* HNPC proliferation. (**A**) Lysates from cultured WT and *RIT1* ^*−*/*−*^ HNPC cultures were analyzed by immunoblotting with the indicated antibodies following IGF-1 stimulation (100 ng/ml; 15 min). Representative images were cropped from the original blots run in parallel. (**B**) Phase contrast and immunocytochemical detection of Nestin (*green*) and DAPI (*blue*) of neurospheres clonally expanded from the dentate gyrus of wild-type mice. (**C**) Proliferation of WT HNPCs was assessed by immunocytochemical detection of Nestin (*green*) and Ki67 (*red*) 24 h following stimulation with or without IGF-1 (50 ng/ml). Scale bar, 15 μm. (**D**) Representative confocal images of transfected Myc-tagged RIT1 (*red*) expression in dissociated WT HNPCs. Scale bar, 10 μm. (**E**) Transfected *RIT1* ^*−*/*−*^ HNPCs (Myc-tagged RIT1 or empty vector) were left untreated or stimulated with IGF-1 (50 ng/ml) and proliferation was assessed at 24 h by immunohistochemical detection of Nestin (*green*) and Ki67 (*red*). Scale bar, 15 μm. (**F**,**G**) Quantification of IGF-1 mediated HNPC proliferation (Nestin^+^/Ki67^+^)/200 cells counted from 5–6 fields in WT (*p < 0.05 nonparametric one tailed t-test), *RIT1* ^*−*/*−*^, and following re-expression of Myc-RIT1 in *RIT1* ^*−*/*−*^ HNPCs (*p < 0.05, one-way ANOVA). Results are presented as mean ± SEM calculated from three separate experiments.

Running exercise increases the availability of several classes of growth factor, including BDNF and IGF-1, which have known roles in regulating adult neurogenesis^30^. While RIT1 plays a role downstream of diverse mitogen-activated receptors^34^, we have previously shown that BDNF signaling in primary hippocampal neuron cultures is not altered by *RIT1* deficiency^36^. In agreement with earlier *in vitro* studies^41^, IGF-1 exposure (100 ng/ml, 15 min) led to robust ERK and Akt activation in wild-type hippocampal cultures (Fig. 2A). Importantly the activation of both kinases was blunted (~55% of kinase phosphorylation of WT hippocampal neuronal cultures, n = 3, p < 0.05) in *RIT1* ^*−*/*−*^ cultures as monitored by anti-phospho-specific immunoblotting (Fig. 2A). Consistent with a role for RIT1 in IGF-1 signaling, wild-type primary hippocampal neural progenitor cells (HNPCs) (Fig. 2B) displayed increased proliferation (p < 0.01) following IGF-1 exposure (Fig. 2C,F), while *RIT1* ^*−*/*−*^ HNPCs failed to respond (p > 0.05) (Fig. 2E,G), as assessed by confocal microscopy (co-stained Nestin^+^/Ki67^+^ cells). Expression of Myc-tagged *RIT1* rescued IGF-1 dependent proliferation in *RIT1* ^*−*/*−*^ HNPCs (p < 0.05) (Fig. 2D,E and G). These data suggest that RIT1 plays a key role in IGF-1 signaling and contributes to HNPC proliferation *in vitro*.

**Figure 3:**
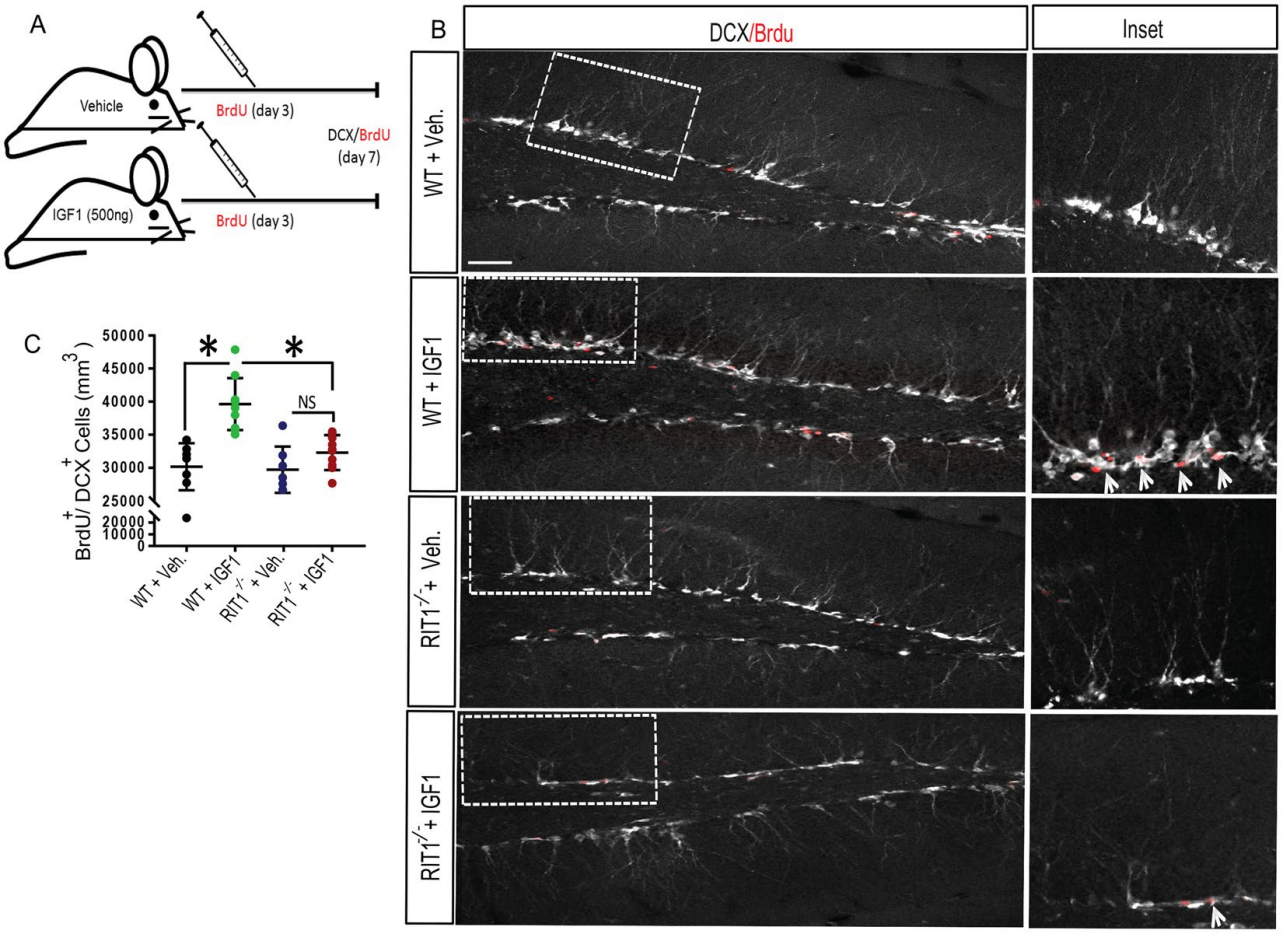
*RIT1* deficiency impairs IGF-1-dependent adult neurogenesis. (**A**) WT and *RIT1* ^*−*/*−*^ mice (n = 10 per genotype) were continuously administered either rhIGF-1 (500 ng/kg/day) or vehicle via subcutaneous pump infusion for 1 week. At day 3 of infusion, mice were i.p. injected with BrdU (50 mg/kg) (×4) over 12 h. (**B**) Representative coronal hippocampal sections co-immunostained for BrdU (*red*) and DCX (*white*) (Scale bar 20 μm). The inserts are at higher magnification. Arrowheads (*white*) indicate newborn DCX^+^/BrdU^+^ neuroblasts. (**C**) Quantification of the BrdU^+^/DCX^+^ proliferating neuroblast density (cell counts/mm^3^) within the dentate gyrus of WT and *RIT1* ^*−*/*−*^ mice (*p < 0.01, one-way ANOVA). Results are presented as mean ± SEM.

We next asked whether RIT1 signaling contributes to IGF-1-dependent *in vivo* stimulation of hippocampal neurogenesis^15^. In agreement with previous studies^15, 16, 18^, peripheral infusion of exogenous recombinant IGF-1 (500 ng/kg/day) (Fig. 3A) was found to induce neurogenesis in the mouse hippocampus (Fig. 3B). Using BrdU labeling, we found a significant increase in newborn BrdU^+^/DCX^+^ immature neurons in the dentate granule cell layer of the hippocampus of WT mice after 7 d of peripheral IGF-1 administration, when compared to vehicle controls (Fig. 3B,C). While vehicle treated WT and *RIT1* ^*−*/*−*^ mice displayed similar numbers of BrdU^+^/DCX^+^ newborn immature neurons, IGF-1 dependent progenitor cell proliferation was significantly blunted in the dentate of *RIT1* ^*−*/*−*^ mice (Fig. 3B,C) (p < 0.05). Taken together, both *in vivo* and *in vitro* data indicate that RIT1 plays a critical role in IGF-1 induced neurogenesis.

### IGF-1 regulates neurogenic transcription factor expression in HNPCs

**Figure 4:**
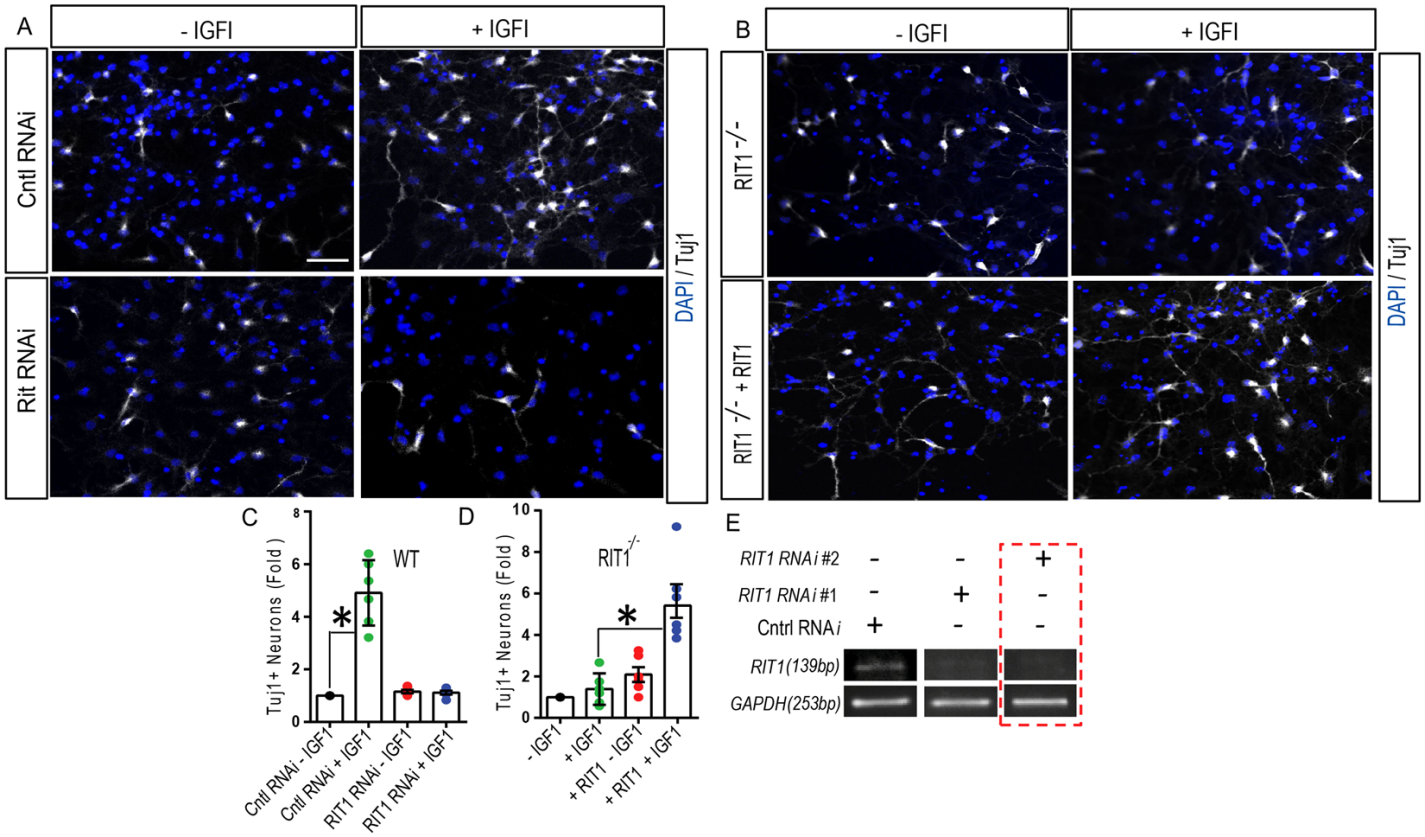
*RIT1* knockdown impairs IGF-1-dependent neural induction of HNPCs. (**A**) WT HNPC cultures were left untreated (-IGF1) or treated with IGF-1 (50 ng/ml, 24 h) following infection with either control (Cntl) or RIT1-RNAi lentivirus and co-immunostained with Tuj1 (*white, pseudo color*) and DAPI (nuclei; *blue*) (Scale bar 10 μm). (**B**) *RIT1* ^*−*/*−*^ HNPCs were transfected with or without Myc-tagged RIT1 and co-immunostained with Tuj1 (*white, pseudocolor*) and for nuclei (DAPI: *blue*) following vehicle or IGF-1 stimulation (50 ng/ml, 24 h). (**C**,**D**) Quantification of Tuj1^+^ cells (n = 500 cells from 5–6 random fields) in HNPCs following either lentiviral-mediated *RIT1* knockdown, or gene rescue in *RIT1* ^*−*/*−*^ HNPCs, with or without IGF-1 stimulation (*p < 0.05, one-way ANOVA). (**E**) RT-PCR analysis of lentiviral-mediated *RIT1* RNAi silencing efficiency in HNPCs. RIT1#2 RNA*i* was used in all subsequent studies.

IGF-1 has been shown to direct the differentiation of hippocampal NPCs to mature granule neurons^17^, and IGF-1 treatment of wild-type HNPC cultures significantly increased the number of Tuj1^+^ neurons (Fig. 4A,C) (p < 0.05). In keeping with a central role for RIT1 in IGF-1 signaling, the ability of IGF-1 to stimulate HNPC neuronal induction was significantly blunted following RNA*i*-mediated *RIT1* silencing (Fig. 4A,C and E), resulting in significantly fewer Tuj1^+^ neurons when compared with HNPC cultures treated with control RNA*i* (p < 0.05). Similar results were seen in cultured *RIT1* ^*−*/*−*^ HNPCs, in which IGF-1 stimulation failed to promote the robust neuronal differentiation observed in WT cultures (Fig. 4B,D). Importantly, the neurogenic deficit in *RIT1* ^*−*/*−*^ HNPCs could be rescued by re-expression of Myc-tagged RIT1 (Fig. 4B and D) (p < 0.05).

**Figure 5:**
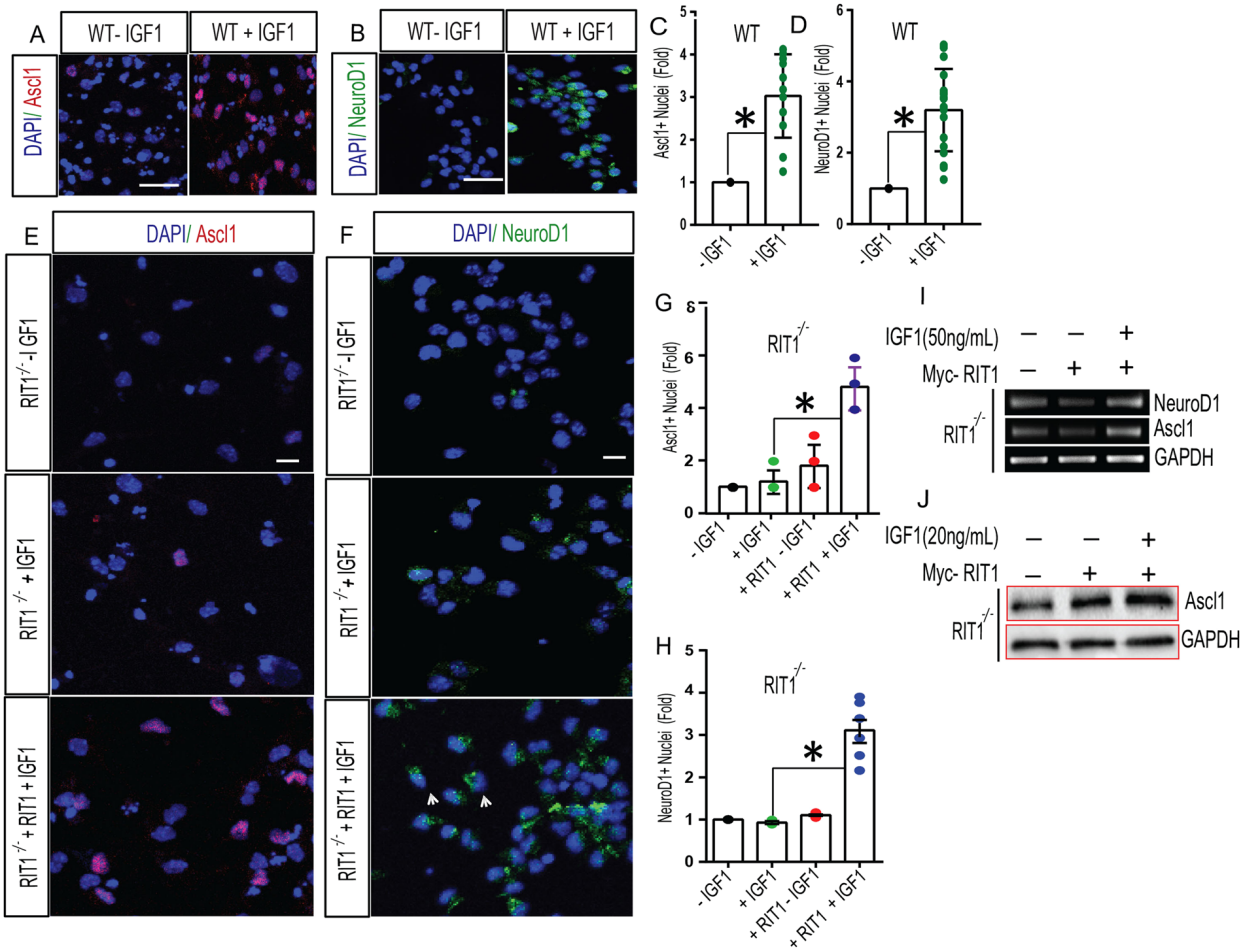
Role for RIT1 in IGF-1-dependent neuronal differentiation. (**A**,**B**) Representative confocal images of Ascl1 (*red*) and NeuroD1 (*green*) expression in WT HNPCs with or without IGF-1 (50 ng/ml, 24 h) stimulation. Scale bar, 20 μm. (**C**,**D**) Quantification (n = 200 cells from 5–6 fields) of Ascl1^+^ or NeuroD1^+^ neural precursor cells in WT HNPCs with or without IGF-1 stimulation (*p < 0.05, nonparametric one tailed t-test). (**E**,**F**) Representative confocal images of Ascl1^+^ (*red*) and NeuroD1^+^ (*green*) cells in *RIT1* ^*−*/*−*^ HNPCs or *RIT1* ^*−*/*−*^ HNPCs following re-expression of Myc-RIT1, with and without IGF-1 stimulation (50 ng/ml, 24 h). Scale bar, 15 μm. (**G**,**H**) Quantification of Ascl1^+^ precursors (*p < 0.05, one-way ANOVA) and NeuroD1^+^ neuronal precursors (*p < 0.05, one-way ANOVA) following RIT1 complementation with or without IGF-1 stimulation. (**I**) Semi-quantitative RT-PCR demonstrates IGF-1-mediated increases in NeuroD1 and Ascl1 expression in *RIT1* ^*−*/*−*^ HNPCs following RIT1 complementation. Representative images of the gene bands were cropped from the original agarose gels. (**J**) Representative Western blot showing RIT1-dependent increase in Ascl1 protein following IGF-1 (20 ng/ml) stimulation of *RIT1* ^*−*/*−*^ HNPCs. Representative images of the protein bands were cropped from the original blots run in parallel. Results are presented as mean ± SEM calculated from three separate experiments.

Adult neurogenesis involves the activation of a multistep transcriptional network^42^ including the sequential expression of Ascl1^43^ and NeuroD1^44, 45^ transcription factors. To determine whether IGF-1 stimulation of HNPCs results in the activation of this transcriptional cascade, we used immunohistochemical analysis to determine the effect of IGF-1 on Ascl1 and NeuroD1 expression in HNPCs. As seen in Fig. 5, IGF-1 stimulation of HNPCs (50 ng/ml IGF-1, 24 h) resulted in a prominent increase in both Ascl1 (Fig. 5A and C) and NeuroD1 (Fig. 5B and D) expressing HNPCs. Consistent with a role for RIT1 in this process, IGF-1 stimulation failed to significantly increase levels of either Ascl1 or NeuroD1 in *RIT1* ^*−*/*−*^ HNPCs (Fig. 5E–H) (p > 0.05), but importantly, Myc-RIT1 re-expression was capable of restoring IGF-1-dependent increases in both transcription factors (Fig. 5E–H) (p < 0.05). Consistent with these data, RT-PCR analysis demonstrates that IGF-1 stimulation leads to an increase in the expression of both NeuroD1 and Ascl1, in a manner that depends upon RIT1 (Fig. 5I). Furthermore, immunoblotting demonstrates a RIT1-dependent increase in Ascl1 protein levels following IGF-1 (20 ng/ml) stimulation in HNPCs (Fig. 5J). Thus, RIT1 deficiency blunts IGF-1-dependent regulation of pro-neurogenic gene expression and neural differentiation of HNPCs.

### IGF-1 regulates SOX2 stability and transcriptional activity in HNPCs

**Figure 6:**
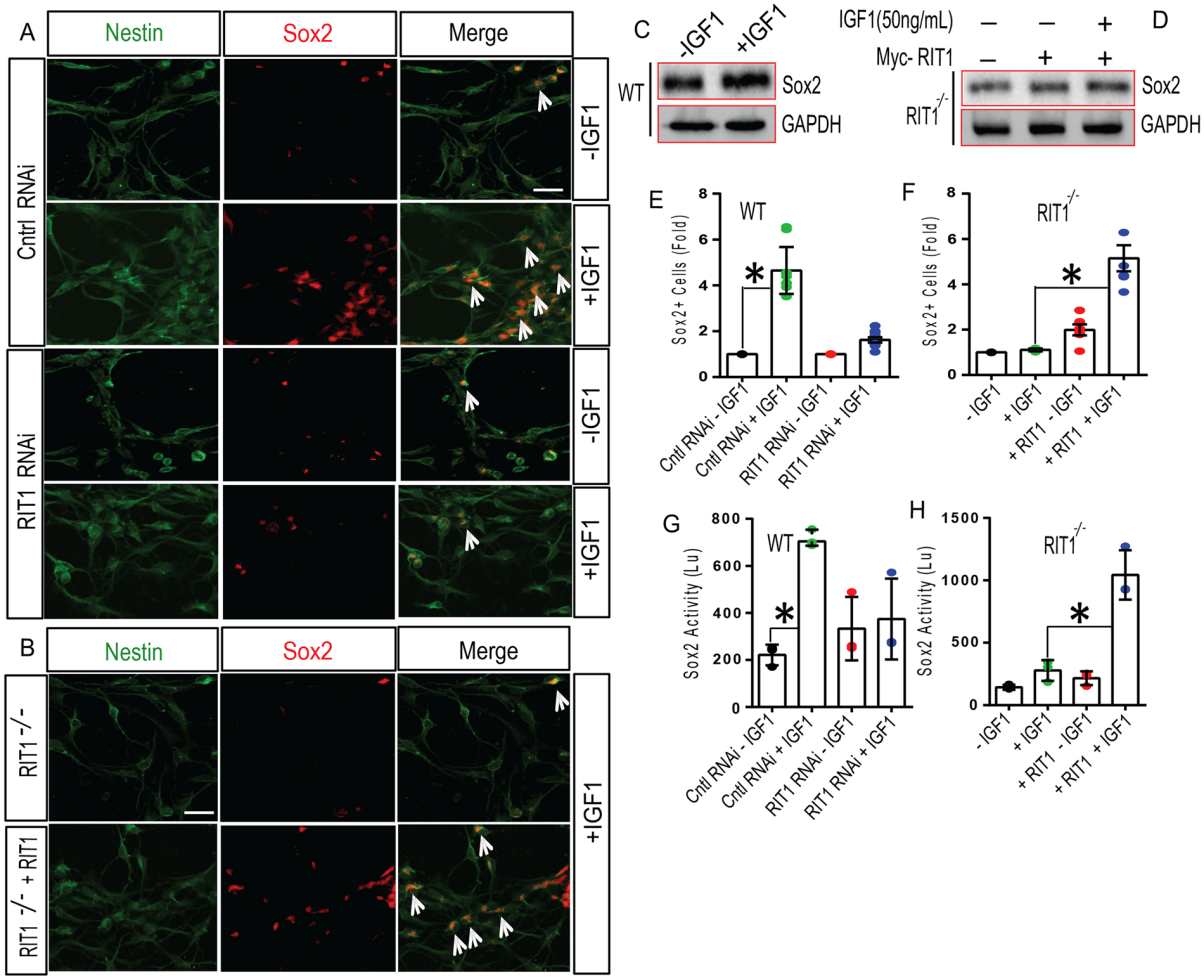
*RIT1* contributes to IGF-1-mediated Sox2 stabilization and transcriptional activation. (**A**) Representative confocal images of Sox2^+^ cells from WT HNPC cultures left untreated (-IGF1) or treated with IGF-1 (50 ng/ml; 24 h) (Scale bar, 15 μm) following infection with either control (Cntl) or *RIT1*-directed RNAi (*RIT1#2*) and co-immunostained with Nestin (*green*) and Sox2 (*red*). The arrowheads (*white*) indicate HNPCs that express Sox2. (**B**) Representative micrographs of Sox2 expression in *RIT1* ^*−*/*−*^ HNPCs, with and without re-expression of Myc-RIT1 following IGF-1 (50 ng/ml; 24 h) stimulation (Scale bar 15 μm). (**C**) Immunoblotting for SOX2 in WT HNPCs with or without stimulation with IGF-1 (50 ng/ml; 24 h). Representative images were cropped from the original blots run in parallel. (**D**) Immunoblotting for SOX2 levels in *RIT1* ^*−*/*−*^ HNPCs in the presence of transiently transfected Myc-RIT1 and IGF-1 stimulation (50 ng/ml; 24 h). Representative images were cropped from the original blots run in parallel. (**E**,**F**) Quantification (fold-change) of Sox2^+^ cells in the indicated HNPC cultures following IGF-1 stimulation (50 ng/ml; 24 h) are presented as mean ± SEM (*p < 0.05, one-way ANOVA). (**G**,**H**) Quantification of Sox2 luciferase reporter activity in cultured WT, *RIT1* ^*−*/*−*^, or *RIT1* ^*−*/*−*^ HNPCs following Myc-RIT1 complementation, with or without IGF-1 stimulation (50 ng/ml, 24 h) are presented as mean ± SEM from three separate experiments (*p < 0.01, one-way ANOVA).

Recent work has shown that the transcription factor SRY (sex determining region Y)-box 2 (*SOX2*), is expressed by adult NSCs, and required both for the maintenance of NPC pluripotency and in establishing the epigenetic state required for neuronal differentiation in response to neurogenic cues^46^. We have recently found that constitutively activate RIT1 is capable of stimulating SOX2-dependent gene expression^39^. To evaluate whether RIT1-SOX2 signaling contributes to IGF-1-dependent neurogenesis, we analyzed the effect of IGF-1 on SOX2 protein levels in HNPCs. As seen in Fig. 6, IGF-1 (50 ng/ml, 24 hrs) stimulation of wild-type HNPCs resulted in a prominent increase in SOX2^+^ cells (p < 0.05) (Fig. 6A,E), and SOX2 protein levels (Fig. 6C). RNA*i*-dependent RIT1 silencing blunted the IGF-1 mediated increase in SOX2 protein levels (Fig. 6A,E) (p < 0.05). A similar result was seen in IGF-1 stimulated *RIT1* ^*−*/*−*^ HNPCs (Fig. 6B,D,F), with re-expression of RIT1 capable of restoring the IGF-1-dependent increase in SOX2 levels (Fig. 6B,D,F) (p < 0.05). Importantly, robust SOX2 transcriptional activity was observed in wild-type HNPCs following IGF-1 stimulation (50 ng/ml) (p < 0.01), but failed to induce SOX2 reporter activity in HNPCs following RNA*i*-mediated RIT1 silencing (Fig. 6G). Similar results were seen in *RIT1* ^*−*/*−*^ HNPCs with the deficit in SOX2 transcriptional activity rescued by re-expression of wild-type RIT1 (p < 0.1) (Fig. 6H). Taken together, these data suggest that IGF-1-dependent hippocampal neurogenesis involves a RIT1-SOX2 signaling cascade.

### IGF-1/RIT1/Akt-dependent SOX2-T118 phosphorylation during neuronal induction

**Figure 7:**
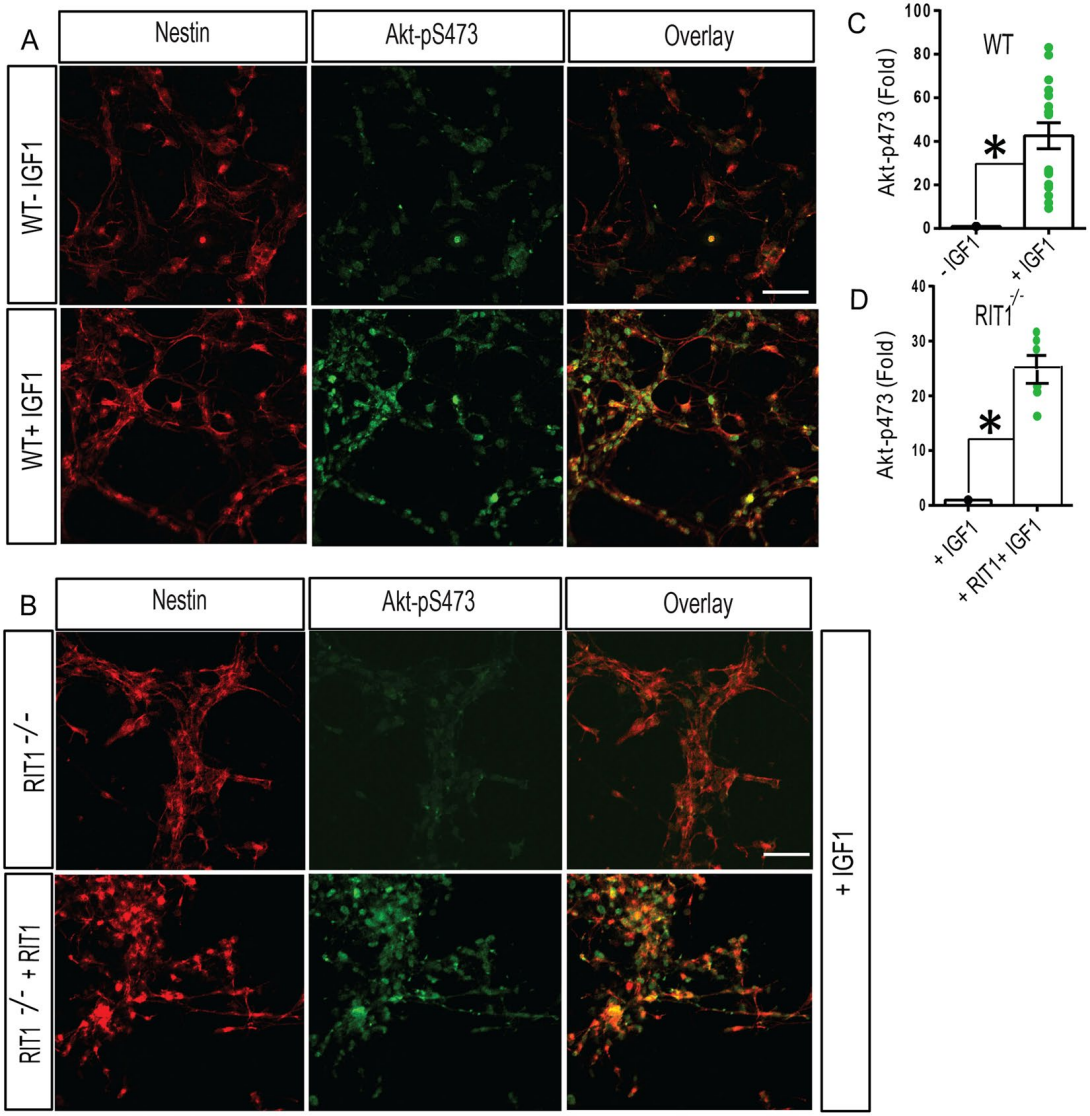
RIT1 contributes to IGF-1-mediated Akt activation. (**A**,**B**) Representative confocal images of WT, *RIT1* ^*−*/*−*^, or *RIT1* ^*−*/*−*^ HNPCs completed by re-expression with Myc-RIT1. HNPC cultures were co-immunostained for phospho-Akt S473 (*green*) and Nestin (*red*) following vehicle or IGF-1 stimulation (50 ng/ml; 24 h). Scale bar, 20 μm. (**C**,**D**) Quantification of phospho-Akt S473 levels following IGF-1 stimulation of the indicated HNPC cultures is presented as mean ± SEM calculated from three separate experiments (*p < 0.01, nonparametric one tailed t-test).

**Figure 8:**
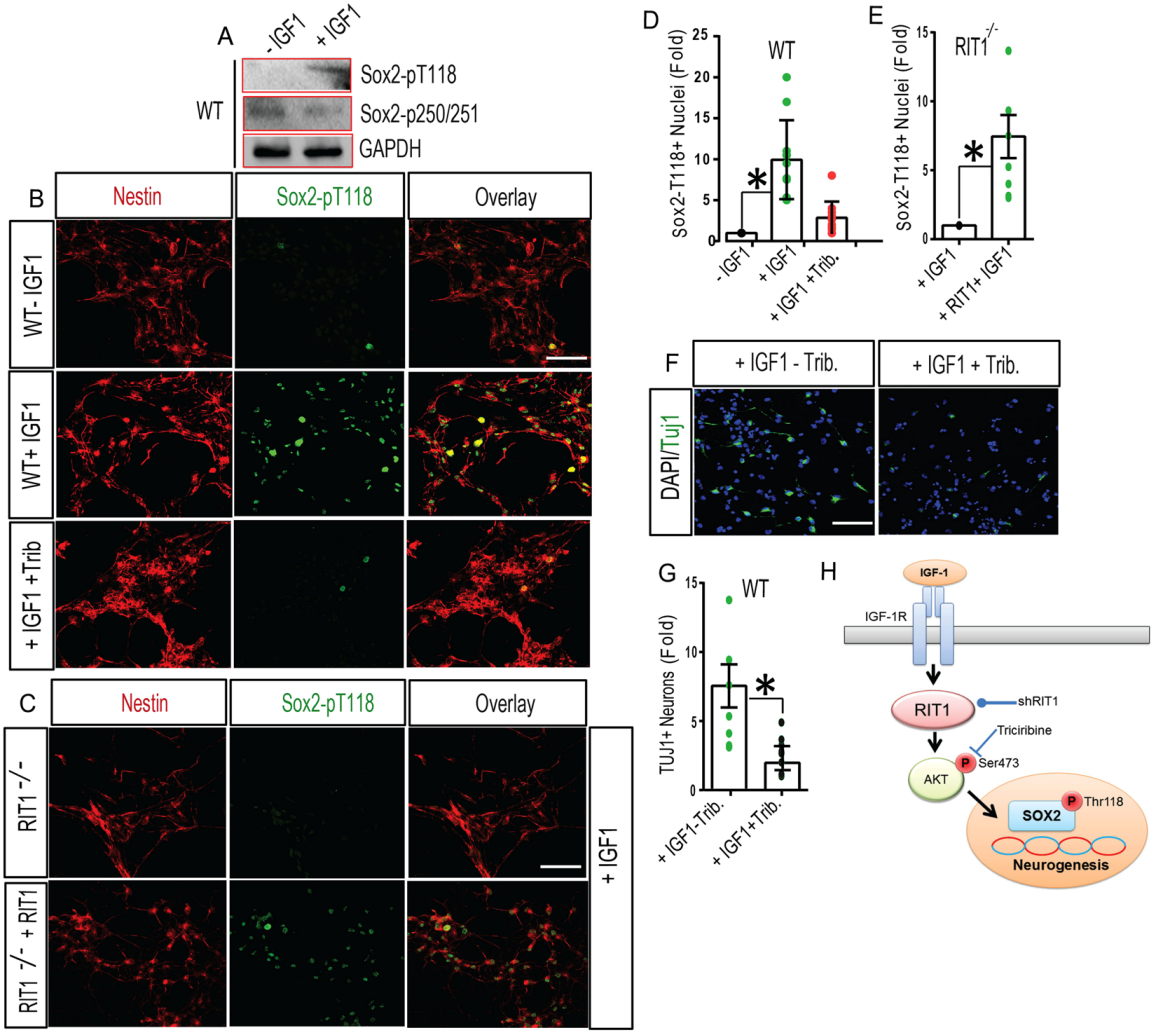
IGF-1-dependent Sox2 phosphorylation relies on *RIT1*-Akt signaling. (**A**) Immunoblotting for levels of Sox2 phospho-Ser250/Ser251 and SOX2 phospho-T118 with and without IGF-1 stimulation (50 ng/ml; 24 h). Representative images were cropped from the original blots run in parallel. (**B**,**C**) Representative confocal images of phospho-Sox2 T118^+^ nuclei in WT, *RIT1* ^*−*/*−*^, or *RIT1* ^*−*/*−*^ HNPCs complemented by re-expression of Myc-RIT1 left untreated (-IGF-1) or 24 h after stimulation with IGF-1 (50 ng/ml) or co-stimulated with IGF-1 and the Akt inhibitor Triciribine (3 µM Trib.) and immunostained for phospho-Sox2 T118 (*green*) and Nestin (*red*). Scale bar, 20 μm. (**D**,**E**) Quantification of Nestin^+^/Sox2 T118^+^ cells following IGF-1 stimulation of WT HNPCs with or without Akt inhibition (1–4 uM Trib.) (*p < 0.05, one-way ANOVA) or IGF-1 stimulation of *RIT1* ^*−*/*−*^ HNPCs with or without RIT1 complementation (*p < 0.05, nonparametric one tailed t-test) (≥200 cells counted from 5–6 fields). (**F**) Representative confocal images of HNPCs co-immunostained for Tuj1^+^ (*green*) and DAPI (nuclei, *blue*) following IGF-1 (50 ng/ml) stimulation with or without Akt inhibition (1–4 uM Trib.). (**G**) Quantification of Tuj1^+^ neurons in WT HNPCs following IGF-1 stimulation (50 ng/ml) ± Triciribine (3 μM) (≥500 cells from 5–6 fields) (*p < 0.05, nonparametric t-test). Data are presented as mean ± SEM calculated from three separate experiments. (**H**) Schematic diagram of the putative IGF-1/RIT1/Akt/Sox2 signal transduction cascade. The sites of pharmacological inhibition (⊥) and the target of shRNA silencing reagents (•) are indicated.

Recent studies have found that SOX2 is regulated by a methylation-phosphorylation switch, in which phosphorylation at T118 stabilizes the SOX2 protein and enhances its transcriptional activity in embryonic stem cells^47^. Whether a similar mechanism operates to control SOX2 during hippocampal neurogenesis is unknown, but since neural expression of active RIT1 promotes SOX2-T118 phosphorylation^39^, we hypothesized that this cascade contributes to IGF-1 dependent SOX2 activation in HNPCs. In support of this notion, we observed IGF-1-dependent stimulation of Akt activity in wild-type HNPCs, as detected by phospho-specific Akt^S473^ immunofluorescence (Fig. 7A,C) (p < 0.01). In agreement with our earlier data (Fig. 2A), *RIT1* ^*−*/*−*^ HNPCs displayed blunted Akt activation (Fig. 7B,D) (p < 0.01) which could be rescued by RIT1 re-expression (Fig. 7B,D). Western blotting found that IGF-1 exposure dramatically increased the phosphorylation of SOX2 at T118 while the phosphorylation at Ser25/Ser251 was unaffected (Fig. 8A). Confocal laser scanning microscopy also showed higher number of phospho-SOX2-T118^+^ nuclei in wild-type HNPCs (Fig. 8B,D) (p < 0.05). Pharmacological blockade of Akt signaling with Triciribine^48^ inhibited the number of phospho- SOX2-T118^+^ nuclei following IGF-1 stimulation (Fig. 8B,D). IGF-1-dependent SOX2-T118 phosphorylation was also blunted in *RIT1* ^*−*/*−*^ HNPCs, and this deficit could be complemented by expression of exogenous RIT1 (Fig. 8C,E). The loss of SOX2 in adult HNPCs has been shown to decrease the expression of pro-neural genes, compromising the differentiation of newborn neurons^46^. Importantly, Akt inhibition significantly reduced the number of Tuj1^+^ neurons in IGF-1 treated HNPC cultures (Fig. 8F,G) (p < 0.01). Taken together, these data indicate that IGF-1 directs a downstream RIT1-Akt-SOX2 signaling cascade to regulate neurogenesis (Fig. 8H).

## Discussion

Studies in the mammalian central nervous system support an essential role for insulin and insulin-like growth factors in stem cell self-renewal, neurogenesis, and cognitive function through distinct ligand-mediated receptor activation cascades^13^. Although IGF-1 has long been associated with the regulation of neural stem cell biology, and much is known about the diversity of IGF-1-dependent signaling cascades^19^, mechanisms by which IGF-1 governs neurogenesis remain incompletely characterized. Here, we provide evidence for the involvement of *RIT1* as a critical downstream component of IGF-1-dependent, and exercise-mediated enhancement, of hippocampal neurogenesis. We demonstrate that, IGF-1 regulates Sox2 activation and neuronal differentiation in a RIT1-dependent fashion in HNPCs, with RIT1 directing IGF-1-mediated expression of the pro-neuronal Ascl1 and NeuroD1 genes, and Akt-dependent up-regulation of Sox2 transcriptional activity. Thus, we demonstrate that RIT1/Akt signaling plays a crucial role in IGF-1 dependent neural cell fate selection by controlling Sox2 function.

During both CNS development and adult neurogenesis, IGF-1/IGF-1R signaling has been found to regulate the proliferation and survival of neuronal progenitors as well as the generation, differentiation, and maturation of neurons^11, 12, 18, 49–51^. Circulating IGF-1 has been implicated in the beneficial effects of exercise on brain function, including increased hippocampal neurogenesis^15, 52^. Previous findings have also identified roles for IGF-1 signaling in neuronal precursor proliferation and neuronal differentiation^24, 53–56^. Our results provide new insight into the molecular mechanisms underlying exercise-enhanced and IGF-1-driven hippocampal neurogenesis. The blunted neurogenic response of exercised *RIT1* ^*−*/*−*^ mice suggests that RIT1 participates in the transduction of running stimuli to promote enhanced hippocampal neurogenesis, whether in response to IGF-1 or another exercise myokine^57, 58^. Not surprisingly, RIT1 deficiency does not result in a complete block of exercise-enhanced neurogenesis (Fig. 1), as neurogenesis is controlled by a broad and sophisticated regulatory network^6^. Indeed, other Ras-related G-proteins are likely involved, as Ras-GRF2, a calcium-regulated exchange factor for Ras and Rac GTPases, has been found to contribute neurogenesis in response to enriched environment^59^. However, the failure of IGF-1 infusion to stimulate neuroblast proliferation in *RIT1* ^*−*/*−*^ mice suggests that RIT1 plays an essential role in IGF-1 dependent regulation of hippocampal neurogenesis, as other Ras family GTPases cannot compensate for *RIT1* loss. While a number of studies have implicated elevated IGF-1 levels as a putative mediator of exercise induced neurogenesis^15, 52^, and our data would support a role for IGF-1/RIT1 in the benefits of exercise on brain plasticity, a growing literature suggests that a variety of factors beyond IGF-1 likely contribute to this process. Indeed, recent studies have identified the exercise myokine, cathepsin B (CTSB)^58^, as an important mediator of the neurogenic benefits of exercise, and additional peripheral blood factors have been shown to improve brain plasticity in aged animals^57^. Therefore, further studies are needed to determine whether RIT1, or other Ras family GTPases, contribute to these signaling pathways.

A growing literature supports a central role for Akt is the regulation of neuronal stem cell proliferation^60, 61^, including the control of exercise-mediated hippocampal neurogenesis^28, 62^ and IGF-1 signaling in neuronal stem cells^22, 41, 63^. Our observation that IGF-1-mediated ERK and Akt activity is blunted in *RIT1* ^*−*/*−*^ HNPC cultures (Fig. 2), but not following BDNF stimulation^36^, suggests that RIT1 deficiency might generate an IGF-1 selective, rather than global, defect in growth factor signaling within the HPNC niche. While these data implicate RIT1 as a key downstream regulator of neuronal IGF-1 signaling, the full contribution of RIT1 to IGF-1 signal transduction remains to be addressed, particularly details on how RIT1 deficiency impacts gene expression. In addition, it is important to identify the guanine nucleotide exchange factor(s) (GEF) involved in coupling IGF-1R activation to stimulation of neuronal RIT1 signaling. This is complicated by the fact that while activation of a SOS/Shc-Grb2 complex has been associated with *in vitro* RIT1 activation following either NGF or PCAP stimulation of pheochromocytoma cells, biochemical analysis has yet to identify a bona fide RIT1GEF. IGF-1 has been shown to be neuroprotective in models of traumatic brain injury^64, 65^, stroke and ischemic injury^66, 67^, preventing apoptotic death and promoting cell survival. As expression of active RIT1 has been shown to be neuroprotective *in vitro* ^37^, studies are underway to determine activated RIT1 signaling is neuroprotective, capable of reducing behavioral deficits in the setting of brain trauma.

Sox2 is involved in the maintenance and proliferation of NPCs *in vivo*, neurosphere formation *in vitro* ^68, 69^, and plays a key role in somatic cell reprogramming^70–73^. Furthermore, voluntary running is known to increase dividing Sox2^+^ HNPCs in the dentate gyrus leading to enhanced neurogenesis^27^. We find potentiation of Sox2 transcriptional activity, stabilization of Sox2 protein levels, and increased proliferation and neural differentiation of HNPCs following IGF-1 stimulation, suggesting that IGF-1/RIT1 regulation of Sox2 may represent a fundamental mechanism for controlling neurogenesis. IGF-1 has been shown to regulate Sox2 in a variety of systems, including human mesenchymal^74^ and colonic stem cells^75^. Thus, it is possible that IGF-1/RIT1 signaling might regulate Sox2 activity in diverse cell populations. We present data indicating that Akt is required for IGF1/RIT1-dependent Sox2 activity. This extends recent studies showing that Akt signaling contributes to the regulation of embryonic stem cell fate by controlling Sox2 stabilization and transcriptional activity by a phosphorylation at threonine 118 (T118)^47^. Consistent with a conserved regulatory mechanism, we find that IGF-1 stimulates Sox2 T118 phosphorylation in HNPCs, resulting in higher levels of Sox2 T118^+^ nuclei (Figs 6 and 8). Using *RIT1* ^*−*/*−*^ HNPCs, RNA*i* methods, and pharmacological Akt inhibition, we demonstrate that RIT1 and Akt are required for IGF-mediated Sox2 transcriptional activation and neuronal differentiation (Figs 6–8). Following traumatic brain injury we have shown that conditional overexpression of IGF-1 leads to increased hippocampal neurogenesis^64, 76^, while post-TBI neurogenesis is delayed in *RIT1* ^*−*/*−*^ mice^36^. Whether these changes rely upon changes in Sox2 function remains to be determined.

Interestingly, there is a growing literature suggesting that IGF-1 is critical for malignant transformation and metastasis of cancer cells^77–79^, and that Sox2 controls tumor initiation and cancer stem-cell function^80, 81^. We have found that conditional *RIT1* overexpression in the dentate gyrus leads to the robust generation of neuroblasts^39^. Recently, we also identified *RIT1* as a novel driver oncogene in a subset of human lung adenocarcinomas, and our data suggest PI3K/Akt inhibition as a potential therapeutic strategy in *RIT1*-mutated tumors^38^. Studies are underway to determine whether oncogenic *RIT1*-dependent tumorigenesis involves activation of Akt-Sox2 signaling.

In summary, we demonstrate a key role for the Ras-related GTPase, *RIT1*, in both IGF-1 and exercise-induced neurogenesis. IGF-1 dependent NPC proliferation and neural differentiation are inhibited by genetic deletion of the *RIT1* gene. We also present data that provides new insight into the mechanisms underlying IGF-1-directed neurogenesis. Our findings reveal that a previously uncharacterized IGF-1/RIT1/Akt/Sox2 signaling cascade is import for regulating the proliferation and differentiation of neural precursors within the dentate gyrus. Collectively, these data provide new insight into mechanisms by which IGF-1 signaling and Sox2 function in neural stem-cell maintenance, embryogenesis and neuronal development.

## Materials and Methods

### Mouse running and BrdU labeling

*RIT1* ^*−*/*−*^ mice have been previously described^36^. 12-week-old male *RIT1* ^*−*/*−*^ mice and their WT littermates were divided into 2 groups and randomly placed in standard-sized rat cages (6 mice per cage) with (running group) or without (sedentary group) 2 (6 inch) running wheels^82^. Wheel running activity for each genotype was monitored 24 h/day and 7 days/week using ClockLab software (Actimetrics, Inc). For lineage labeling experiments, each mouse independent of housing was intraperitoneally (i.p.) injected with BrdU (50 mg/kg, in 0.9% Saline) once daily for the first 14 days of the experiment. Newborn immature neuron assessment was performed 2 days after the last BrdU injection (day 16). Neural maturation was quantified 1 month after the last BrdU injection (day 42). Following the either length of housing (16 or 42 days) mice were anesthetized with sodium pentobarbital (65 mg/kg, i.p.), transcardially perfused with heparinized saline followed by 10% buffered formalin, and decapitated. After 24 h of post-fixation in 10% buffered formalin, brains were removed from the skull, post-fixed for an additional 24 h, cryoprotected in 30% sucrose, and quickly frozen in isopentane. Serial coronal 40 µm sections were cut using a freezing sliding microtome (Dolby-Jamison). Every tenth section (400 µm intervals between sections) was selected as a set for further analysis. All experimental procedures were approved by the University of Kentucky Institutional Animal Care and Use Committee in accordance with guidelines established by the National Institutes of Health in the *Guide for the Care and Use of Laboratory Animals*. Animals were housed at up to 5 mice per cage in the University of Kentucky Medical Center vivarium with a 14:10-hour light/dark photoperiod and were provided food and water ad libitum.

### Subcutaneous IGF-1 infusion

Osmotic pumps (ALZET) were filled aseptically with 0.2-µm-filter sterilized saline or rIGF-1 (National Hormone and Peptide Program, Torrance, CA). 12-week-old RIT1^*−*/*−*^ mice (n = 25) and their wild-type littermates were randomly divided into 2 groups (IGF-1 or control), anesthetized with isoflurane, and implanted subcutaneously dorsally^16^ for 7 days. The infusion rate of rIGF-1 was 500 ng/kg/day. At day 3 of infusion, mice were i.p. injected with BrdU (50 mg/kg) at 3 h intervals for 12 h.

### Hippocampal neuronal stem cell (HNPC) cultures

Primary HNPC cultures were prepared as describe^39^. HNPCs were isolated from wild-type and *RIT1* ^*−*/*−*^ mice as described^83^. Briefly, mice were euthanized, the brain dissected and placed in immersion buffer (HBSS (1x) with no Ca^2+^ or Mg^2+^ containing 1x antibiotic solution (Gibco)). Using a stereomicroscope, dentate gyrus (DG) from hippocampi were dissected and placed in ice-cold immersion buffer. DG (4–5/genotype) were washed with HBSS (1x) containing antibiotic, incubated at 37 °C for 30–45 min with frequent shaking in enzymatic digestion solution (0.25% trypsin in 1 × HBSS with activated papain), trypsin activity was quenched by repeated washing with DMEM (5–10 ml), and placed in 37 °C culture medium (containing DMEM/F12 (1:1), supplemented with 0.3% B27 without insulin, 20 ng/ml of EGF and 10 ng/ml bFGF, and antibiotics). HNPCs were released by trituration (3–4 times) into single cells using fire polished Pasteur pipettes. Approximately, 50 × 10^4^ cells were plated in 12 well plates for suspension culture. Neurospheres were evident by day 3. For passage, neurospheres were pooled and mechanically dissociated into single cells and seeded into suspension in growth media in presence of EGF and bFGF (see above). For immunocytochemistry and immunoblotting, single cell suspensions derived from neurospheres were plated on poly D-Lysine coated coverslips or 6 well plates. HNPCs used in this study were Nestin^+^ (HNPC lineage) and >2 passages which promotes homogeneity in the cell population.

### RNA*i*-mediated silencing in HNPCs

Lentiviral vector pZIP-mCMV containing the *RIT1* pri-shRNA sequence (TGCTGTTG ACAGTGAGCGACACGAAGTTCGGGAGTTTAAATAGTGAAGCCACAGATGTATTTAAACTCCCGAACTTCGTGGTGCCTACTGCCTCGGA) was purchased from transOMIC Technologies (Huntsville, AL). Lentivirus was generated in 293LTV cells using the packaging vectors PsPAX2 and pMD2.G (Univ. Kentucky Genetic Technology Core). The efficiency of RNA*i* silencing in HNPCs was determined to be >70% using RT-PCR and confocal microscopy. Briefly, HNPCs after passage were allowed to assume normal morphology for 24–48 h before RNA*i* silencing. Growth medium was removed and stored as per our previously described method^84^, 1 µl of polybrene (Santa Cruz Biotechnology) was added in 1x HBSS for 10 min at 37 °C followed by repeated washes in 1x DPBS. Growth medium was premixed with 3 MOIs of either non-targeting control or RIT1-RNA*i* expressing lentivirus and placed on cells for 4–12 h at 37 °C. Cells were allowed to recover in fresh growth medium for 48–72 h to permit *RIT1* silencing.

### Cell transfection and neuronal differentiation

For HNPC transfection, fresh neurospheres were dissociated and approximately 10^5^ cells were plated on poly-D-lysine coated coverslips/6 well plates. Cells were transfected 72 h after plating, using Jetprime transection reagent^85^ according to the manufacturer’s protocol, and allowed to recover cells 48 h before analysis. For IGF-1 stimulation of HNPCs, DMEM was supplemented with F12 (1:1) and FGF2 (10 ng/ml). For HNPC neuronal differentiation analysis, cells were placed in Neurobasal medium containing 1% FBS and RA (1 µM) for 4–6 days.

### Immunofluorescence

Immunofluorescence or immunocytochemistry was performed as per our previously published method^84^. Mice were anesthetized with sodium pentobarbital (65 mg/kg, i.p.), transcardially perfused with 0.98% saline followed by 4% paraformaldehyde, and decapitated^84^. Brains were removed from the skull, subject to post-fixation (2–3 days) at 4 °C in 4% paraformaldehyde, subject to increasing concentrations of sucrose (10–30%: O/N for each 10% step), and dissected under a stereomicroscope for hippocampus. Tissue blocks were embedded in OCT and snap frozen. All the blocks were stored at −80 °C for at least 2 days before coronal sections (15 µM or 40 µM) were cut using a cryostat, and mounted on superfrost plus slides (Fisher). Antigen retrieval was performed in citrate pH 6.2. For BrdU, antigen retrieval was performed with warm trypsin (0.25%) containing 2N HCl for 5 min at RT. The sections were extensively washed with PBS and incubated in blocking and permeablizing buffer [1% serum (matching the secondary antibody host), 1% Triton X-100 in 1 × PBS) for 10 min at RT followed by extensive washing (4 × PBS). After blocking, primary antibodies were diluted in blocking buffer (0.01 M PBS pH 7.2 containing 1% serum, 0.05% Tween 20 and 0.1% Triton X-100) and applied to the sections O/N (4 °C). Dilutions were: rabbit anti-DCX (1:500); NeuN (1:3000), Rat anti-BrdU (Sigma, 1:800), Sox2 (Abcam, 1:300), Goat NeuroD1 (Santa Cruz, 1:200), Rabbit Nestin (Covance, 1:300), Rabbit Aktp473 (Cell Signaling Technology, 1:400) and Mouse Sox2pT118 (ECM Biosciences, 1:200). On the following day, sections were washed with 1 × PBS (x4) and secondary antibody (goat-anti-rabbit IgG FTIC: 1:1,000; either conjugated with Alexa 488, Alexa 568, Alexa 594 or PE) diluted in blocking buffer was applied for 2 h in the dark. Sections were then washed with 1 × PBS (x4), air dried, and cover slipped with SlowFade Gold containing DAPI. For immunocytochemistry, cells were fixed in 4% paraformaldehyde for 15 minutes at RT, permeablized, and blocked as described for tissue sections. Representative images were captured using a Nikon CKX31 A1 confocal microscope or Nikon C2 confocal microscope. All images were acquired using the NIS Elements software package (University of Kentucky License).

### Image acquisition and analysis

Analysis was performed as previously described^86^. Briefly, isotype control slides were mounted to nullify nonspecific signals from either the Nikon C2 confocal or Nikon A2 confocal prior to mounting non-primary antibody treated slides and channels were adjusted to the individual secondary fluorophore. The numerical aperture and intensity values were optimized to minimize oversaturation and the channel data, pin hole details were recorded, and images recorded at 1024 pixels and 16x speed (Nikon C2 Confocal). Groups of images (3–6 images per specimen) were analyzed using ImageJ. For example, alternate color masks were applied to determine double positive (Nestin^+^/Ki67^+^) or single positive (Nestin^+^ only) for ≥200 nuclei from 5–6 random fields (for normalization), and data converted to relative fold changes. A similar approach was used to determine neuronal differentiation and the number Sox2^+^ and Sox T118^+^ HNPCs. For Akt activation following IGF-1 stimulation, the intensity profile of ≥50 cells from 15 random fields were normalized to DAPI using ImageJ. The mean was determined from 5–8 images per group. Quantification of immature (DCX^+^/BrdU^+^) and mature neuron analysis (NeuN^+^/BrdU^+^) was performed as previously described^36^. Briefly, to quantify the fluorescently labeled cells in the dentate gyrus, three sections (pre-epicenter, epicenter, post-epicenter, 400 μm intervals) were counted using a Nikon Eclipse E600 fluorescence microscope (40× objective). The focal plane was moved throughout the z-axis to capture each positive cell. To estimate the volume of the dentate gyrus, images were collected using a Zeiss Axiovert 200 M fluorescence microscope (10× objective). The volume of the dentate gyrus was then calculated by multiplying the area of the dentate gyrus measured using ImageJ (NIH) by the thickness of each section (40 μm). Cell density was obtained by dividing total cell counts by the total volume of the dentate gyrus for the three sections that were counted.

### Western blotting

Tissues/cells were homogenized using a Next Advance Bullet Blender at 4 °C in lysis buffer (20 mM Tris·HCl pH 7.5, 250 mM NaCl, 10 mM MgCl_2_, 1% Triton X-100, 1 mM Na_3_VO_4_, 50 mM β-glycerophosphate, 1x protease inhibitor cocktail). Whole cell lysates were resolved on SDS-PAGE gels, transferred to nitrocellulose membranes (12 h, 0.08 mA), and protein abundance and phosphorylation determined by immunoblotting with the appropriate phospho-specific antibodies and band intensity quantified using a ChemiDoc MP with Image Lab software (Bio-Rad)^35, 36^.

### Luciferase gene reporter assay

Cignal Sox2 luciferase reporter was obtained from Qiagen (CCS-0038L). HNPCs were transfected with reporter and transduced with RNAi (Control RNA*i* or RIT1 RNA*i*), while *RIT1* ^*−*/*−*^ HNPCs were co-transfected with the reporter and Myc-RIT1 (as indicated) or empty vector, and stimulated with/without IGF-1 (50 ng/ml, 24 h) in presence of FGF2 (10 ng/mL). Cells were washed with PBS and luciferase activity determined using a firefly luciferase assay kit (Promega) after passive lysis as described^39^.

### Statistical analysis

The data is presented as Mean ± SEM. Statistical analysis was carried out by either one way/two way ANNOVA combined with *post hoc* analysis using Tukey Kramer multiple comparisons or nonparametric unpaired one tailed t-test. Significance reported in this manuscript is p < 0.05. Any changes with p > 0.05 were considered to be insignificant.
